# Does age influence the quality of life in children with atopic dermatitis?

**DOI:** 10.1371/journal.pone.0224618

**Published:** 2019-11-14

**Authors:** Milena Ražnatović Đurović, Janko Janković, Vesna Tomić Spirić, Milijana Relić, Zorica Sojević Timotijević, Anđa Ćirković, Slađana Đurić, Slavenka Janković

**Affiliations:** 1 Clinic of Dermatology and Venereology, Clinical Center of Montenegro, Faculty of Medicine University of Montenegro, Podgorica, Montenegro; 2 Institute of Social Medicine, Faculty of Medicine, University of Belgrade, Belgrade, Serbia; 3 Department of Internal Medicine, Faculty of Medicine, University of Belgrade, Belgrade, Serbia; 4 Clinic for Allergology and Immunology, Clinical Center of Serbia, Belgrade, Serbia; 5 Department of Dermatology, Faculty of Medicine, University of Priština, Kosovska Mitrovica, Serbia; 6 Institute of Medical Statistics and Informatics, Faculty of Medicine, University of Belgrade, Belgrade, Serbia; 7 Department for Preventive Medicine, Faculty of Medicine, University of Priština, Kosovska Mitrovica, Serbia; 8 Institute of Epidemiology, Faculty of Medicine, University of Belgrade, Belgrade, Serbia; Clinic for Infectious and tropical diseases, Clinical centre of Serbia, SERBIA

## Abstract

**Background:**

Atopic dermatitis (AD) is one of the most common childhood skin diseases that can affect the quality of life (QoL) of children. The QoL of Montenegrin children with AD has not been sufficiently explored. The aim of this study was to assess their QoL with special emphasize on age differences.

**Methods:**

This cross-sectional study included children with AD seen at the Clinic of Dermatology and Venereology, Clinical Center of Montenegro (CCM) in Podgorica between August 2017 and July 2018. The QoL was assessed with the Infants’ Dermatitis Quality of Life Index (IDQOL) and the Children’s Dermatology Life Quality Index (CDLQI). Disease severity was measured by the Three Item Severity (TIS) score.

**Results:**

A total of 386 children with AD aged from newborn to 16 years took part in this study. The mean total score of the QoL was 14.7 in infants (0–4 years old), 19.4 in younger children (5–9 years old), and 16.6 in older children (10–16 years old). Age was in negative correlation with the CDLQI score, leisure domain of the CDLQI and CDLQI sleep, and in positive correlation with the IDQOL child mood. TIS score was in positive correlation with both the IDQOL and CDLQI score.

**Conclusions:**

The QoL measured by CDLQI was more impaired in younger children, whilst IDQOL child mood was more impaired in older infants. The most impaired QoL was seen in children in the age group 5–9 years. Regardless of disease severity, treatment and counseling of children suffering from AD should be tailored specifically to their age.

## Introduction

Atopic dermatitis (AD) is a chronic inflammatory pruritic skin disorder that affects children and adults. Since the last decade of the previous century, AD has become a significant public health problem because of its increasing prevalence especially in developed regions [1–3]. There is an evidence that up to 20% of children in developed countries suffer from AD [1,4]. However, the prevalence of AD is also increasing in developing regions, including Africa and the Middle East [5]. As a consequence, this condition has a great burden on healthcare resources of societies [6–8]. AD can cause significant morbidity in the affected children and can impair their quality of life (QoL) and the QoL of their families [1,9–12]. Childrens’ perception of QoL may depend on disease severity [13–15], but also on socio-demographic variables such as age [16,17] and gender [17,18].

The QoL of Montenegrin children with AD, especially the association between child’s age and QoL, has not been sufficiently explored. The aim of this study was to assess the QoL of Montenegrin children with AD and to evaluate if age affects their QoL.

## Materials and methods

The cross-sectional study was carried out between August 2017 and July 2018 at the Clinic of Dermatology and Venereology, Clinical Center of Montenegro (CCM) in Podgorica.

Inclusion criteria were consecutive patients aged 0 to 16 years with diagnosis of AD, as formed according to the Hanifin and Rajka diagnostic criteria [19] and absence of other skin-related conditions that could have influenced the study outcomes. A few parents (2%) refused their children to participate in the study.

Ethical approval was obtained from the Ethics Committee of the CCM. Written informed consent was obtained from parents.

Data such as child’s age, sex, presence of other atopic disease and family history of atopy were collected using a short questionnaire.

QoL of children was assessed with the Infants’ Dermatitis Quality of Life Index (IDQOL) for children below the age of 5 years [20] and the Children’s Dermatology Life Quality Index (CDLQI) for children from 5 to 16 years of age [21]. Both questionnaires consist of ten items each, and all questions are related to the week preceding the testing. The items in IDQOL explore itching (question 1), child’s mood (question 2), sleep issue (questions 3 and 4), play time (question 5), family activities (question 6), meal time (question 7), treatment (question 8), dressing (question 9), and bath time (question 10). The items in CDLQI assess symptoms and feelings (questions 1 and 2), leisure (questions 4, 5, and 6), school or holidays (question 7), personal relationships (questions 3 and 8), sleep (question 9) and treatment (question 10). All items in each questionnaire are scored on a 4-point scale from 0 to 3 with higher values indicating more severe impact of AD, resulting in a cumulative score from 0 to 30 for each questionnaire. The higher the score, the more QoL is impaired. Additionally, while filling the IDQOL questionnaire parents were asked to rate the severity of child’s AD from 1 (none) to 4 (extremely severe). Both questionnaires are linguistically validated and culturally adapted from English to Serbian language [22,23].

Disease severity was evaluated using the Three Item Severity (TIS) score which use three of the intensity items of the SCORing Atopic Dermatitis (SCORAD) index: erythema (0–3), oedema (0–3), and excoriations (0–3) in one or several different representative areas with the maximum score of 9. Based on the TIS, the severity of AD was classified into mild (<3), moderate (3–5) and severe (≥6). We decided to use TIS as an easy and fast method which highly correlated with the SCORAD [24,25].

### Statistical analysis

Continuous variables were expressed as mean ± standard deviation (SD) and categorical variables through frequency and percentage. To assess differences between variables χ^2^ test, Student’s t-test, one way ANOVA and Kruskal-Wallis test were used where appropriate. Correlation between IDQOL and CDLQI scores and AD severity (TIS score) was assessed using Pearson’s correlation coefficients.

Multiple linear regression analyses (twenty five models) were used to determine whether IDQOL/CDLQI scores were related to age independently. The dependent variables were overall IDQOL and CDLQI scores, all items of both questioners and CDLQI domain scores. Independent variables in all models were: age, sex, presence of atopic disease, family history of atopy and disease severity (TIS score). They were chosen according to the published literature [10,16,18].

Cronbach’s alpha was applied to assess the reliability of IDQOL and CDLQI questionnaires.

A two-tailed probability value of 0.05 or less was considered significant. All statistical analyses were performed with the Statistical Package for the Social Sciences (SPSS), version 20.0 for Windows (SPSS Inc., Chicago, IL, USA).

## Results

A total of 386 children with AD, aged from newborn to 16 years took part in this study. Characteristics of the study sample are presented on Table 1.

**Table 1:** Characteristics of the study sample according to age groups.

Variable	All ages	0–4 years	5–9 years	10–16 years	*P*
**Number of patients (%)**	386 (100)	186 (48.2)	38 (9.8)	162 (42.0)	
**Age(yr) (mean ± SD)**	7.19 ± 5.11	2.35 ± 1.01	7.47 ± 1.20	12.67 ± 1.94	
**Gender**, n (%)					
Male	150 (38.9)	72 (38.7)	10 (26.3)	68 (42.0)	0.204*
Female	236 (61.1)	114 (61.3)	28 (73.7)	94 (58.0)	
**Atopy**, n (%)					
AD alone	212 (54.9)	134 (72.0)	10 (26.3)	68 (42.0)	<0.001*
Concomitant atopic disease^‡^	174 (45.1)	52 (28.0)	28 (73.7)	94 (58.0)	
**Age at onset** (mean ± SD)	1.29 ± 1.46	0.49 ± 0.66	1.47 ± 1.06	2.14 ± 1.70	**<0.001**^§^
**AD duration** (mean ± SD)	5.98 ±4.44	1.88 ± 0.99	6.00 ± 1.47	10.55 ± 2.27	
**Family history of atopy**, n%					
Yes	262 (67.9)	107 (57.5)	26 (68.4)	129 (79.6)	<0.001*
No	124 (32.1)	79 (42.5)	12 (31.6)	33 (20.4)	
**TIS** (mean ± SD)	4.85 ± 1.31	4.58 ± 1.59	5.16 ± 1.20	5.09 ± 0.82	**<0.001**^†^
**TIS score**, n (%)					
Mild	14 (3.6)	14 (7.5)	0	0	0.003*
Moderate	304 (78.8)	140 (75.3)	32 (84.2)	132 (81.5)	
Severe	68 (17.6)	32 (17.2)	6 (15.8)	30 (18.5)	

*χ^2^ test;

^†^One way Anova;

^‡^Asthma, allergic rhinitis, and/or allergic conjunctivitis;

^§^Kruskal-Wallis test;

AD, Atopic Dermatitis; TIS, Three-Item Severity score.

According to their age, children were divided into three groups: infants (0–4 years old) with mean age 2.35, younger children (5–9 years old) with mean age 7.47 and older children (10–16 years old) with mean age 12.67. There were 61% of girls and 40% of boys. The three groups did not differ according to gender. However, they differed in terms of the presence of the concomitant allergic diseases, family history of atopy and severity of the AD. Concomitant allergic diseases and family history of atopy were more prevalent in children in both groups compared with infants. The mean age at onset of disease and disease duration for children in our sample were 1.29 and 5.98 years, respectively. The mean TIS score of the total sample was 4.85 ± 1.31. TIS score was higher in both older groups of children in comparison with the youngest age group (<0.001). The most prevalent form of AD in our study was a moderate form (78.8%), followed by severe (17.6%) and mild AD form (3.6%).

QoL scores for all items according to age groups were presented in Table 2.

**Table 2:** Quality of life scores according to age groups.

IDQOL		CDLQI			
0–4 years			5–9 years	10–16 years	P*
Item	Mean ± SD	Item	Mean ± SD	Mean ± SD	
**1. Itch**	1.80 ± 0.74	**1. Itch**	2.32±0.66	2.19±0.71	0.352
**2. Mood**	1.83 ± 0.80	**2. Embarrassment**	1.79±1.07	1.56±1.12	0.259
**3. Time to sleep**	1.20 ± 0.91	**3. Friendships**	2.00 ± 0.80	1.82 ± 0.86	0.261
**4. Sleep disturbance**	1.03 ± 0.86	**4. Clothes/shoes**	1.79 ± 0.78	1.55 ± 0.91	0.124
**5. Playing**	1.42 ± 0.84	**5. Leisure/hobbies**	2.10 ± 0.73	1.80 ± 0.81	**0.036**
**6. Family activities**	1.46 ± 0.89	**6. Swimming/sports**	2.10 ± 0.80	1.42 ± 1.06	**<0.001**
**7. Mealtimes**	1.42 ± 0.85	**7. School/holidays**	2.42 ± 0.60	2.24 ± 0.66	0.110
**8. Treatment**	1.72 ± 0.84	**8. Teasing/bullying**	1.95 ± 0.77	1.61 ± 0.80	**0.021**
**9. Dressing**	1.38 ± 0.84	**9. Sleep**	1.47 ± 0.69	1.14 ± 0.88	**0.027**
**10. Bath time**	1.46 ± 0.90	**10. Treatment**	1.42 ± 0.76	1.24 ± 0.71	0.183

IDQOL, Infants’ Dermatitis Quality of Life Index; CDLQI, Children’s Dermatology Life Quality Index;

*t-test.

The highest scoring items of the IDQOL were child’s mood, itching and problems with treatment. The items with the lowest score were the items about sleep disturbance and the time it takes to fall asleep. Questions with the highest CDLQI scores in both age groups (5–9 and 10–16 years of age) were questions related to problems during school/holidays and itching, while the items with the lowest scores were questions about problems with sleep and treatment. The most significant difference between two groups was noticed for item swimming/sports where higher impairment of QoL was gained by children from the age group 5–9 years. Items teasing/bullying, sleep problems, and problems in doing hobbies also had higher values in the age group 5–9 years than in the older age group (10–16 years).

**Fig 1:**
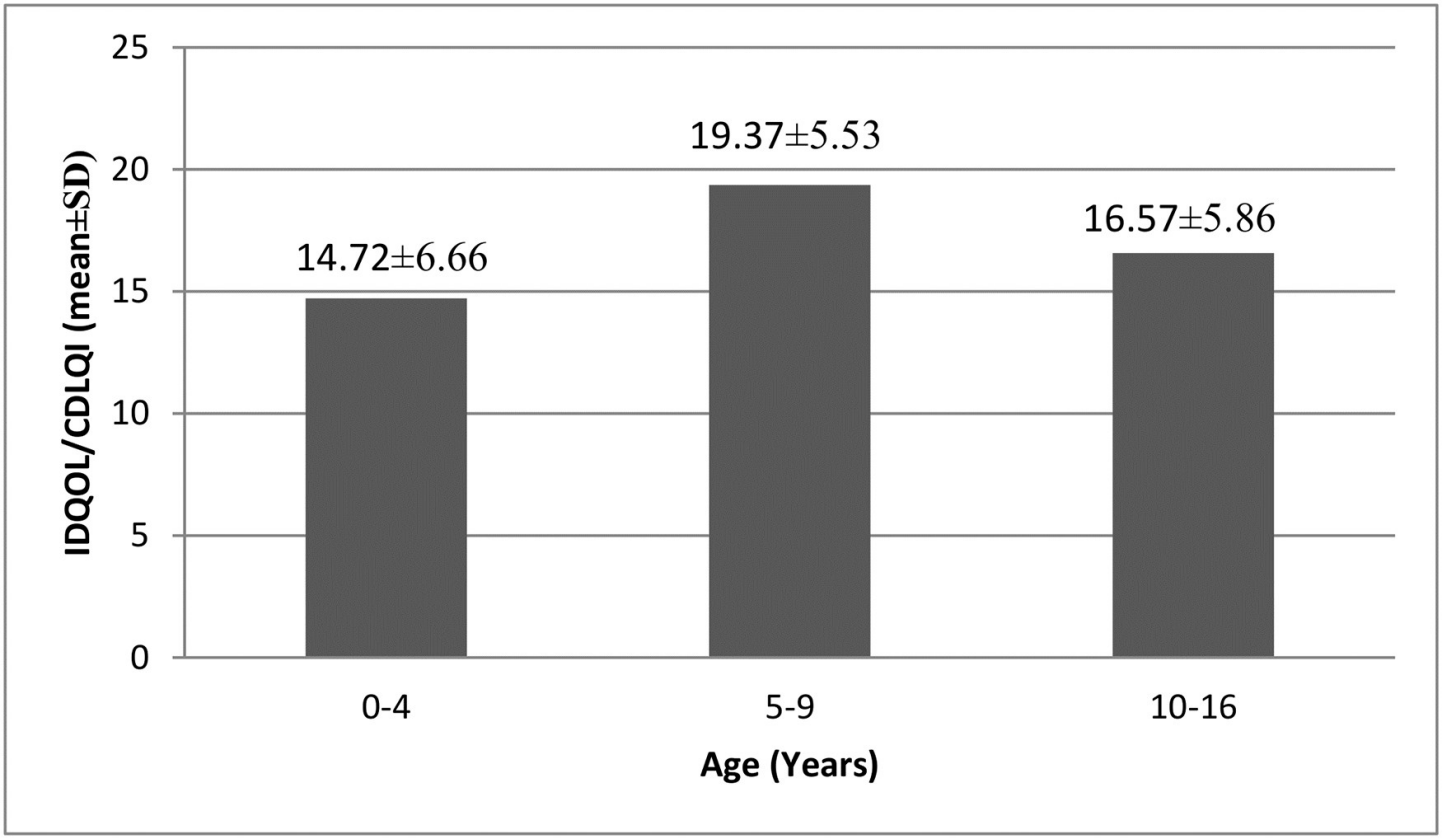
Quality of life scores in different age groups. IDQOL, Infants’ Dermatitis Quality of Life Index; CDLQI, Children’s Dermatology Life Quality Index.

Fig 1 illustrates a significant difference (p <0.001) between the mean total score of the QoL in three age groups. The highest score was seen in children 5–9 years old (19.37) and the lowest in the youngest age group (14.72).

Table 3 presents the correlation between IDQOL/CDLQI and AD severity (TIS score) according to age groups. The strongest correlation was observed between CDLQI and TIS score in the age group 5–9 years (r = 0.84), and moderate correlation in two other age groups (r = 0.61 in the youngest age group; r = 0.46 in the oldest age group). The correlation between overall CDLQI score and the total TIS score was also moderate (r = 0.54).

**Table 3:** Correlation between quality of life (IDQOL/CDLQI) and severity of atopic dermatitis (TIS), according to age groups.

	Age groups				
	0–4	5–9	10–16	5–16	All ages
Coefficient*	0.61	0.84	0.46	0.54	0.59

IDQOL, Infants’ Dermatitis Quality of Life Index; CDLQI, Children’s Dermatology Life Quality Index; TIS, Three-Item Severity score;

*Pearson’s correlation coefficient.

IDQoL/CDLQI mean scores in relation to AD severity (TIS categories) were statistically different (p<0.001). The highest score was seen in the group with severe AD (Table 4).

**Table 4:** Quality of life scores in relation to severity of atopic dermatitis.

	AD severity (TIS score)			*P*
	Mild	Moderate	Severe	
**IDQOL/CDLQI** (mean ± SD)	8.57 ± 3.80	14.86 ± 5.41	22.32 ± 6.35	**<0.001**

AD, Atopic Dermatitis; TIS, Three-Item Severity score; IDQOL, Infants’ Dermatitis Quality of Life Index; CDLQI, Children’s Dermatology Life Quality Index.

According to multiple linear regressions (Table 5), higher IDQOL child mood score was found in older infants, whilst higher overall CDLQI score, CDLQI leisure domain score, and CDLQI items scores clothes/shoes, swimming/sports and sleep, were found in younger children.

**Table 5:** IDQOL and CDLQI items significantly related to age according to multiple linear regression analyses^†^.

Variable	IDQOL Child mood	CDLQI				
		Leisure domain	Clothes/shoes	Swimming/sports	Sleep	Total score
**Age, years**	0.16**	-0.16*	-0.17*	-0.16*	-0.16*	-0.13*

^**†**^Adjusted on sex, disease severity, concomitant atopic disease, and family history of atopy;

IDQOL, Infants’ Dermatitis Quality of Life Index; CDLQI, Children’s Dermatology Life Quality Index;

*P< 0.05;

**P< 0.01.

All the items of both the IDQOL index and CDLQI index showed excellent internal consistency with mean Cronbach’s alpha coefficients of 0.93 and 0.88, respectively.

## Discussion

Our study provided a comparison of QoL impairment in Montenegrin children with AD from different age groups. The most impaired QoL was seen for children 5–9 years old (mean score was 19.37 ± 5.53), followed by QoL for those 10–16 and 0–4 years old (mean score was 16.57 ± 5.86 and 14.72 ± 6.66, respectively). According to the results of multiple regression analyses, only the IDQOL item child mood positively correlated with infants’ age whilst total CDLQI score, CDLQI leisure domain score and CDLQI item scores clothes/shoes, swimming/sports and sleep negatively correlated with children’s age.

Only several studies investigated association between child’s age and QoL in patients with AD. Ganemo et al. [16] found that the mean CDLQI score was higher for the younger children than the older ones (8.38 + 3.99 for 5–8 years of age vs.6.38 + 4.49 for 9–15 years). In the international multi-center study conducted by Chernyshov et al. [26] data on the CDLQI questionnaire of 167 AD children 5–16 years old from Ukraine, Czech Republic, Singapore, and Italy were used for the study. Only in Czech children the overall CDLQI score positively correlated with their age. Other studies did not find any significant association between child’s age and QoL in AD subjects [10,27,28].

Current study showed that not all aspects of QoL are affected equally in observed age groups. The most negative effect on QoL with AD in our study was related to child’s mood, itching and treatment in the youngest age group (0–4 years old) and problems during school/holidays, itching and leisure/hobbies in both older age groups (5–9 and 10–16 years of age). Concerns about itching had the second highest mean score in all age groups that is in accordance with previous studies in which itching had very high or even the highest impact on the children’s QoL [21,26,29–31]. Itch or pruritis, defined as an unpleasant sensation that provokes the desire to scratch, is a major characteristic and one of the most disabling symptoms in allergic and atopic diseases [32]. It is an essential diagnostic feature of AD [19,33] with the potential to severely compromise QoL. It is well known that nocturnal scratching might considerably impair sleep and cause fatigue and irritability [34]. Sleep is disrupted in up to 60% of children with eczema, increasing to 83% during exacerbation [35]. Yosipovitch et al. [36] found that 84% of the AD patients reported difficulty falling asleep, with 79% reported being awakened by pruritis. Reid and Lewis-Jones [37] suggested that children with AD lose an average of 2 hours of sleep per night. Although sleep disturbance was not among the most affected QoL issues in our study, the mean score of this item was much higher than in most previous studies [29,30]. Sleep deprivation leads to tiredness, mood changes and impaired psychosocial functioning of the child including effects on school performance and daily social and leisure activities [31]. Along with the affected children, other family members may also suffer as a result of being awakened [38].

In our study items swimming/sports, teasing/bullying, sleep problems and problems in doing hobbies had higher values in the age group 5–9 years than in the older age group (10–16 years). According to Hon et al. [17], itch and sleep disturbance were the specific areas that particularly troubled younger children (5–10 years of age vs. 11–16 years).

Embarrassment, teasing and bullying frequently cause social isolation and may lead to depression or school avoidance [31]. In our study treatment difficulties were found to be the predominant problems in infants that is in agreement with other studies [30,31].

In addition, we have shown a strong correlation between both QoL measurement tools (IDQOL/CDLQI) and clinical disease severity that is in agreement with previous studies [10,26,27,29,39–43].

The main strength of this study is a large sample size of patients with AD. However, some degree of caution should be exercised when interpreting the results, because the study was performed in a single dermatology clinic of a teaching hospital where more severe patients used to be treated thus it may not be possible to extrapolate the findings to primary care. Our sample included patients with a wide range of AD severities, with a majority of moderate and severe cases. It is unlikely that the results will remain the same in different disease severities. One of the limitations of this study is the assessment of QoL because a high proportion of study participants are too young to provide information about their own QoL and that parents/caregivers are asked to estimate QoL of their children.

## Conclusions

In conclusion, the present study illustrates that age besides disease severity is an important factor in QoL issue. The most impaired QoL in Montenegrin children was seen in the age group 5–9 years in which the strongest correlation between QoL and AD severity was observed. More impaired QoL of affected children was associated with more severe AD. Regardless of disease severity, treatment and counseling of children suffering from AD should be tailored specifically to their age.

## Supporting information

S1 File Dataset. (XLSX)

## References

[1] Deckers IA, McLean S, Linssen S, Mommers M, van Schayck CP, Sheikh A. Investigating international time trends in the incidence and prevalence of atopic eczema 1990–2010: a systematic review of epidemiological studies. PLoS One. 2012;7(7):e39803 10.1371/journal.pone.0039803 22808063 PMC3394782

[2] Jackson KD, Howie LD, Akinbami LJ. Trends in allergic conditions among children: United States, 1997–2011. NCHS Data Brief. 2013;121: 1–8. 23742874

[3] Hansen TE, Evjenth B, Holt J. Increasing prevalence of asthma, allergic rhinoconjunctivitis and eczema among schoolchildren: Three surveys during the period 1985–2008. Acta Paediatr. 2013;102: 47–52. 10.1111/apa.12030 22994385

[4] Williams H, Stewart A, von Mutius E, Cookson W, Anderson HR, ISAAC Phase One and Three Study Groups. Is eczema really on the increase worldwide? J Allergy Clin Immunol. 2008;121: 947–954. 10.1016/j.jaci.2007.11.004 18155278

[5] Al-Afif KAM, Buraik MA, Buddenkotte J, Mounir M, Gerber R, Ahmed HM, et al Understanding the Burden of Atopic Dermatitis in Africa and the Middle East. Dermatol Ther (Heidelb). 2019;9: 223–241. 30850961 10.1007/s13555-019-0285-2 PMC6522619

[6] Verboom P, Hakkaart-Van L, Sturkenboom M, De Zeeuw R, Menke H, Rutten F. The cost of atopic dermatitis in the Netherlands: an international comparison. Br J Dermatol. 2002;147: 716–724. 10.1046/j.1365-2133.2002.04964.x 12366418

[7] Mancini AJ, Kaulback K, Chamlin SL. The socioeconomic impact of atopic dermatitis in the United States: a systematic review. Pediatr Dermatol. 2008;25: 1–6. 10.1111/j.1525-1470.2007.00572.x 18304144

[8] Carroll CL, Balkrishnan R, Feldman SR, Fleischer AB Jr, Manuel JC. The burden of atopic dermatitis: impact on the patient, family, and society. Pediatr Dermatol. 2005;22: 192–199. 10.1111/j.1525-1470.2005.22303.x 15916563

[9] Chamlin SL, Frieden IJ, Williams ML, Chren MM. Effects of atopic dermatitis on young American children and their families. Pediatrics. 2004;114: 607–611. 10.1542/peds.2004-0374 15342828

[10] Monti F, Agostini F, Gobbi F, Neri E, Schianchi S, Arcangeli F. Quality of life measures in Italian children with atopic dermatitis and their families. Ital J Pediatr. 2011;37: 59 10.1186/1824-7288-37-59 22192570 PMC3287107

[11] Amaral CS, March Mde F, Sant’Anna CC. Quality of life in children and teenagers with atopic dermatitis. An Bras Dermatol. 2012;87: 717–723. 10.1590/s0365-05962012000500008 23044564

[12] Marciniak J, Reich A, Szepietowski JC. Quality of Life of Parents of Children with Atopic Dermatitis. Acta Derm Venereol. 2017;97: 711–714. 10.2340/00015555-2633 28207075

[13] Holm EA, Wulf HC, Stegmann H, Jemec GB. Life quality assessment among patients with atopic eczema. Br J Dermatol. 2006;154: 719–725. 10.1111/j.1365-2133.2005.07050.x 16536816

[14] Boccardi D, D’Auria E, Turati F, DI Vito M, Sortino S, Riva E, et al Disease severity and quality of life in children with atopic dermatitis: PO-SCORAD in clinical practice. Minerva Pediatr. 2017;69: 373–380. 10.23736/S0026-4946.16.04294-8 26200523

[15] Ng MS, Tan S, Chan NH, Foong AY, Koh MJ. Effect of atopic dermatitis on quality of life and its psychosocial impact in Asian adolescents. Australas J Dermatol. 2018;59:e114–e117. 10.1111/ajd.12632 28836265

[16] Ganemo A, Svensson A, Lindberg M, Wahlgren CF. Quality of life in Swedish Children with Eczema. Acta Derm Veneorol. 2007;87: 345–349. 10.2340/00015555-0245 17598039

[17] Hon KL, Leung TF, Wong KY, Chow CM, Chuh A, Ng PC. Does age or gender influence quality of life in children with atopic dermatitis? Clin Exp Dermatol. 2008;33: 705–709. 10.1111/j.1365-2230.2008.02853.x 18681872

[18] Chernyshov PV, Ho RC, Monti F, Jirakova A, Velitchko SS, Hercogova J, et al Gender Differences in Self-assessed Health-related Quality of Life in Children with Atopic Dermatitis. J Clin Aesthet Dermatol. 2016;9: 19–24. PMC5022992 27672414

[19] Hanifin JM, Rajka G. Diagnostic features of atopic dermatitis. Acta Derm Venereol (Stockh). 1980;92: 44–47.

[20] Lewis-Jones MS, Finlay AY, Dykes PJ. The Infants’ Dermatitis Quality of Life Index. Br J Dermatol. 2001;144: 104–110. 10.1046/j.1365-2133.2001.03960.x 11167690

[21] Lewis-Jones MS, Finlay AY. The Children’s Dermatology Life Quality Index (CDLQI): initial validation and practical use. Br J Dermatol. 1995;132: 942–949. 10.1111/j.1365-2133.1995.tb16953.x 7662573

[22] Cardiff University, School of Medicine. The Infant’s Dermatitis Quality of Life Index (IDQOL)—different language versions—Serbian version. https://www.cardiff.ac.uk/medicine/resources/quality-of-life-questionnaires/infants-dermatitis-quality-of-life-index [Accessed 10 September 2019].

[23] Cardiff University, School of Medicine. The Children’s Dermatology Life Quality Index (CDLQI)—different language versions—Serbian version. https://www.cardiff.ac.uk/medicine/resources/quality-of-life-questionnaires/childrens-dermatology-life-quality-index [Accessed 10 September 2019].

[24] Wolkerstorfer A, de Waard-van der Spek FB, Glazenburg EJ, Mulder PG, Oranje AP. Scoring the severity of atopic dermatitis: three item severity (TIS) score as a rough system for daily practice and as a prescreening tool for studies. Acta Derm Venereol. 1999;79: 356–359. 10.1080/000155599750010256 10494710

[25] Oranje AP, Glazenburg EJ, Wolkerstorfer A, de Waard-van der Spek FB. Practical issues on interpretation of scoring atopic dermatitis: the SCORAD index, objective SCORAD and the three-item severity score. Br J Dermatol. 2007;157: 645–648. 10.1111/j.1365-2133.2007.08112.x 17714568

[26] Chernyshov PV, Ho RC, Monti F, Jirakova A, Velitchko SS, Hercogova J, et al An international multicenter study on self-assessed and family quality of life in children with atopic dermatitis. Acta Dermatovenerol Croat. 2015;23: 247–253. 26724875

[27] Maksimović N, Janković S, Marinković J, Sekulović LK, Zivković Z, Spirić VT. Health-related quality of life in patients with atopic dermatitis. J Dermatol. 2012;39: 42–47. 10.1111/j.1346-8138.2011.01295.x 22044078

[28] Jiráková A, Vojáčková N, Göpfertová D, Hercogová J. A comparative study of the impairment of quality of life in Czech children with atopic dermatitis of different age groups and their families. Int J Dermatol. 2012;51: 688–692. 10.1111/j.1365-4632.2011.05175.x 22607286

[29] Ben Gashir MA, Seed PT, Hay RJ. Quality of life and disease severity are correlated in children with atopic dermatitis. Br J Dermatol. 2004;150: 284–290. 10.1111/j.1365-2133.2004.05776.x 14996099

[30] Beattie PE, Lewis-Jones MS. A comparative study of impairment of quality of life in children with skin disease and children with other chronic childhood diseases. Br J Dermatol. 2006;155: 145–151. 10.1111/j.1365-2133.2006.07185.x 16792766

[31] Lewis-Jones S. Quality of life and childhood atopic dermatitis: the misery of living with childhood eczema. Int J Clin Pract. 2006;60: 984–992. 10.1111/j.1742-1241.2006.01047.x 16893440

[32] Buddenkotte J, Steinhoff M. Pathophysiology and therapy of pruritus in allergic and atopic diseases. Allergy. 2010;65: 805–821. 10.1111/j.1398-9995.2010.01995.x 20384615

[33] Darsow U, Pfab F, Valet M, Huss-Marp J, Behrendt H, Ring J, et al Pruritus and atopic dermatitis. Clin Rev Allergy Immunol. 2011;41: 237–244. 10.1007/s12016-010-8230-2 21207193

[34] Arndt J, Smith N, Tausk F. Stress and atopic dermatitis. Curr Allergy Asthma Rep. 2008;8: 312–317. 18606083 10.1007/s11882-008-0050-6

[35] Camfferman D, Kennedy JD, Gold M, Martin AJ, Lushington K. Eczema and sleep and its relationship to daytime functioning in children. Sleep medicine reviews. 2010;14: 359–369. 10.1016/j.smrv.2010.01.004 20392655

[36] Yosipovitch G, Goon AT, Wee J, Chan YH, Zucker I, Goh CL. Itch characteristics in Chinese patients with atopic dermatitis using a new questionnaire for the assessment of pruritus. Int J Dermatol. 2002;41: 212–216. 10.1046/j.1365-4362.2002.01460.x 12031029

[37] Reid P, Lewis-Jones MS. Sleep difficulties and their management in preschoolers with atopic eczema. Clin Exp Dermatol. 1995;20: 38–41. 10.1111/j.1365-2230.1995.tb01280.x 7671394

[38] Misery L, Finlay AY, Martin N, Boussetta S, Nguyen C, Myon E, et al Atopic dermatitis: impact on the quality of life of patients and their partners. Dermatology. 2007;215: 123–129. 10.1159/000104263 17684374

[39] Kiebert G, Sorensen SV, Revicki D, Fagan SC, Doyle JJ, Cohen J, et al Atopic dermatitis is associated with a decrement in health-related quality of life. Int J Dermatol. 2002;41: 151–158. 10.1046/j.1365-4362.2002.01436.x 12010340

[40] Kim DH, Li K, Seo SJ, Jo SJ, Yim HW, Kim CM, et al Quality of life and disease severity are correlated in patients with atopic dermatitis. J Korean Med Sci. 2012;27: 1327–1332. 10.3346/jkms.2012.27.11.1327 23166413 PMC3492666

[41] Alzolibani AA. Impact of atopic dermatitis on the quality of life of Saudi children. Saudi Med J. 2014;35: 391–396. 24749137

[42] Ražnatović Djurović M, Janković J, Tomić Spirić V, Janković S. Health-related Quality of Life in Children with Moderate to Severe Atopic Dermatitis. Acta Dermatovenerol Croat. 2015;23: 178–184. 26476901

[43] Chernyshov PV, Jirakova A, Ho RC, Moed H, Caldeira AP, Alvarenga TM, et al An international multicenter study on quality of life and family quality of life in children with atopic dermatitis. Indian J Dermatol Venereol Leprol. 2013;79: 52–58. 10.4103/0378-6323.104669 23254729

